# Smartphone Apps for Pulmonary Hypertension: Systematic Search and Content Evaluation

**DOI:** 10.2196/57289

**Published:** 2024-10-30

**Authors:** Nerea Báez Gutiérrez, Héctor Rodríguez Ramallo, Elva María Mendoza-Zambrano, Berenice Brown Arreola, Bernardo Santos Ramos, Laila Abdel-kader Martín, Remedios Otero Candelera

**Affiliations:** 1 Pharmacy Department, Hospital Universitario Virgen Macarena, Seville, Spain; 2 Pharmacy Department, Hospital Universitario Virgen del Rocio, Avenida Manuel Siurot, S/n, Seville, 41013, Spain, 34 955 01 20 95; 3 Instituto de Biomedicina de Sevilla, Universidad de Sevilla, Hospital Universitario Virgen del Rocío, Seville, Spain

**Keywords:** pulmonary hypertension, mobile apps, smartphone, eHealth, mHealth, app, hypertension, chronic condition, mobile health app, monitoring, systematic search, app development, clinical validation, evaluation, pulmonary

## Abstract

**Background:**

Pulmonary hypertension (PH) is a chronic and complex condition, requiring consistent management and education. The widespread use of smartphones has opened possibilities for mobile health apps to support both patients and health care professionals in monitoring and managing PH more effectively.

**Objective:**

This study aimed to identify and assess the quality of free smartphone apps for PH targeted at either patients or health care professionals.

**Methods:**

A systematic search was conducted on freely available apps for patients with PH and health care professionals, accessed from a Spanish IP address, on Android (Google Play) and iOS (App Store) platforms. Searches were performed in October 2022 and 2023. Apps were independently analyzed by two reviewers, focusing on general characteristics. Quality assessment was based on the Mobile Application Rating Scale (MARS) framework, and Mann-Whitney *U* tests compared mean MARS scores against specific variables.

**Results:**

In the overall study, 21 apps were identified. In the 2022 search, 19 apps were listed (9 iOS, 7 Android, 3 available on both platforms). In the subsequent 2023 search, 16 apps were identified (6 Android, 7 iOS, 3 available on both platforms). Of those identified in 2022, 14 remained available in 2023, with only 7 updated since 2022. In addition, 12 apps targeted patients or the general population, while 9 targeted health care professionals; none involved patients in the development or design. Conversely, 13 apps involving health care professionals were identified. There were 10 apps that received pharmaceutical industry funding. The primary goal for 81% (17/21) of the apps was to disseminate general information about PH. The overall mean MARS quality was acceptable in 2022 and 2023, with mean ratings of 3.1 (SD 0.6) and 3.3 (SD 0.5), respectively. The functionality category achieved the highest scores in both years, indicating ease of use and intuitive navigation. In contrast, the subjective quality domain consistently received the lowest ratings in the MARS assessment across both years. None of the apps underwent clinical testing themselves; however, 2 incorporated tools or algorithms derived from trials. The overall quality of iOS apps statistically outperformed that of Android apps in both years (*P*<.05). Furthermore, the involvement of health care professionals in app development was associated with enhanced quality, a trend observed in both years (*P*=.003 for both years).

**Conclusions:**

This review of mobile health apps for PH reveals their emergent development stage, with generally acceptable quality but lacking refinement. It highlights the critical role of health care professionals in app development, as they contribute significantly to quality and reliability. Despite this, a notable stagnation in app quality and functionality improvement over 2 years points to a need for continuous innovation and clinical validation for effective clinical integration. This research advocates for future app developers to actively engage with health care professionals, integrate patient insights, and mandate rigorous clinical validation for PH management.

## Introduction

Pulmonary hypertension (PH) is a pathophysiological disorder encompassing various clinical conditions, often associated with cardiovascular and respiratory diseases [1]. Effective management of PH requires a comprehensive, multidisciplinary approach within specialized centers, ensuring enhanced patient care through collaborative diagnosis, treatment, and continuous quality monitoring [1,2]. Regular communication between patients, their informal caregivers, and the health care professionals involved, including those external to the primary treatment team, is vital for effective disease management [3,4]. This communication enhances patient adherence to the prescribed therapy and ensures meticulous monitoring [5,6]. In this context, mobile health (mHealth) technologies, particularly smartphone apps, present a novel opportunity to enhance disease management. These apps have become pivotal resources for either health care professionals or patients in managing and monitoring diseases such as PH [7,8].

Although the integration of mHealth technologies could expand patient care in PH, it raises significant questions regarding the willingness of patients and health care professionals to invest financially in these digital health tools. Research exploring the willingness to pay for health management apps among these groups is limited [9-12]. Findings suggest a general reluctance among patients to spend money on such apps [9,11,13], likely influenced by the widespread availability of free alternatives that offer similar functionalities. This reluctance underscores the importance of focusing on these no-cost options, reflecting the economic realities and preferences of most patients and health care providers.

Assessing the quality of these free health apps is crucial in order to identify deficiencies and opportunities for improvement and address concerns regarding the accuracy of medical information, user privacy, and accessibility—particularly for populations with limited technology access [14,15]. This analysis can guide the development of future apps and provide valuable insights to health care professionals and patients, aiding them in the selection of more effective and safer apps for health management. However, it is important to acknowledge that the specific application of these technologies in PH may still need to be explored [16].

Currently, our knowledge about the number and quality of free apps for caregivers and patients with PH, as well as the variation in their availability and quality across years, is limited. Therefore, this cross-sectional study aims to conduct a thorough evaluation of commercially available free PH apps, targeted at either patients or health care professionals, for 2 consecutive years. This analysis will focus on the characteristics and overall quality of these apps, addressing the identified gaps in evidence and providing insights into their development and evolution.

## Methods

### Study Design

This study was designed to evaluate the quality and availability of free smartphone apps for PH targeted at either patients or health care professionals over 2 consecutive years, 2022 and 2023. The decision to conduct the study across these 2 years was based on the dynamic nature of mHealth technologies, where rapid advancements and frequent updates are common. By analyzing the apps over this period, we aimed to capture changes, improvements, and trends that might occur within a relatively short time frame, providing a comprehensive view of the current state and evolution of PH-related mHealth apps.

### Search Strategy

A systematic search of mHealth apps specifically relating to PH was conducted using a Spanish IP address on the iOS (App Store) and Android (Google Play) platforms. The initial search was performed on October 5, 2022, with a follow-up search on October 10, 2023, to compare the evolution of apps over 1 year. The search terms used were “pulmonary hypertension,” “pulmonary,” “lung,” “SEPAR” (Sociedad Española de Patología Respiratoria), “European Respiratory Society,” “ERS,” “European Society of Cardiology,” “ESC,” and, when applicable, their equivalents in the Spanish language.

The following inclusion criteria were used for the selection of apps:

- Apps targeted at patients with PH
- Apps targeted at health care professionals providing care for patients with PH
- Apps that included general information about PH

The following exclusion criteria were used:

- Apps that were not freely available
- Apps that were not available in English and/or Spanish
- Apps providing information exclusively about human anatomy
- Apps providing exclusively quizzes or trivia about a disease
- Apps that included inappropriate content (eg, horoscopes, astrology)
- Apps aimed exclusively at fundraising
- Apps with nonfunctional links

The apps had to comply with at least 1 inclusion criteria and none of the exclusion criteria to be included in the data extraction phase.

The search was executed on mobile devices, reflecting a real user’s approach to finding and downloading apps. This method ensures the selection of apps that are directly accessible and functional for end users, aligning with the typical user experience on smartphones and tablets.

The names and descriptions of the apps from the search in the Google Play Store and the Apple App Store were screened against predefined selection criteria. The apps that met the inclusion criteria were downloaded for further screening using a smartphone (Android version 13) for Google Play Store apps. For the apps downloaded from the App Store, an iPad Pro 2020 (Apple Inc) running iOs iPadOS 15.1 was used. For apps available on both platforms, the iOS version was selected for evaluation due to the relatively smaller number of apps on this platform, a method consistent with established methodologies in previous studies [17-19]. This approach not only streamlined the analysis within the confines of our resource constraints but also was supported by literature indicating minimal variability between the platforms in terms of usability and overall quality scores [20-23].

### Data Extraction

Data were obtained from the online app descriptions (including app characteristics and the narrative text) found on the App Store and Google Play Store by 2 independent researchers. Data were extracted and entered into a structured Microsoft Excel 2021 database (Microsoft Corp).

Variables collected for each app were the following: name, developer/owner name, type of developer/owner (eg, commercial, scientific societies, patient associations), platform (Android or iOS), language (English and/or Spanish), app store category (ie, education, health and fitness, medicine), date of publication (year), date of the last update (year), participation of health care professionals in the design and/or development of the apps (yes/no), and participation of patients in the design/development of the apps.

For the purposes of this study, an update was defined as any new version of an app released by its developers, which may include improvements in functionality, user interface changes, additional features, bug fixes, or any other modifications aimed at enhancing the app.

The involvement of health care professionals was acknowledged if specified in the app’s description or if health organizations, such as scientific societies and hospitals, developed the app. Patient participation was noted when explicitly mentioned in the app’s description.

Technical aspects of the apps were also documented, including features such as social media sharing capabilities (eg, Facebook, Twitter), the requirement of a login, and the necessity of web access for functionality. Additionally, information was collected on whether the apps had received funding or sponsorship from the pharmaceutical industry, and on their potential commercial interests, including the presence of advertisements or options for purchasing services.

The purposes of the apps were classified into the following categories: assessment (ie, apps providing clinical scales, classifying adverse event severity, interpretation for laboratory findings), general information (ie, apps providing information about PH, medication, adverse events), and monitoring of clinical parameters (ie, apps providing a register of laboratory parameters, symptoms). Apps aimed at patients or caregivers were also classified into the following categories: register of patient activities (ie, apps providing calendars for patients to add appointments and treatment administration) and contact with health care professionals.

A descriptive analysis was developed, with continuous and discrete variables presented as mean (SD) and n (%), respectively. The means of continuous variables were compared using the Mann-Whitney *U* test. Results with a *P* value <.05 were considered statistically significant. The data generated were analyzed using IBM SPSS Statistics (version 26.0; IBM Corp).

### App Quality Evaluation

To evaluate app quality, the Mobile Application Rating Scale (MARS) was used [24]. The MARS is a validated system to assess health apps, which has been described as the most comprehensive for evaluating technical information and capabilities of apps [24-26]. This tool has been widely used to evaluate health apps designed for many diseases [17,27-29].

The MARS comprises 23 items across 5 subscales:

- Engagement: evaluates the entertainment, interest, customization, interactivity, and adequacy of the target group.
- Functionality: assesses the performance, ease of use, navigation, and gestural design of the app.
- Aesthetics: examines the layout, graphics, and visual appeal of the app.
- Information: assesses the accuracy of the description, establishment of goals, quality and quantity of information, visual information, credibility of the source, and evidence-based development of the app.
- Subjective quality: determines willingness to recommend the app, times the apps will be used, willingness to pay, and overall rating of the app.

Each criterion was evaluated from 1 to 5 (1=inadequate; 2=poor; 3=acceptable; 4=good; 5=excellent). A mean score of the 5 subscales was calculated to describe overall quality.

Mean scores of the engagement, functionality, aesthetics, and information quality objective subscales were calculated, and an overall mean app quality total score was determined. Mean scores were used instead of total scores to accommodate items rated as “Not applicable” and align with familiar star rating formats. The subjective quality items were scored separately or averaged into a mean subjective quality score. This separation ensured that the inherently subjective nature of these measures, based solely on the evaluators’ personal opinions and without any predefined criteria, did not unduly influence the overall objective quality assessment. This approach provides a clearer distinction between subjective perceptions and objective functionality and content quality [24].

Before the app search was carried out, reviewers read and became acquainted with the MARS tool. All authors then discussed each rating criterion to achieve a consensus on how to apply it. The first app included was evaluated concurrently by all reviewers to ensure a common understanding and application of the MARS tool. Subsequently, each app included in the study was independently evaluated by 2 reviewers. The scores of each item from these independent evaluations were then compared. In cases where the initial assessments aligned, the average of these scores was calculated to establish the overall mean app quality total score. In instances of significant discrepancy between the 2 primary reviewers’ scores—defined as a difference greater than 1 point—where a consensus could not be reached through discussion, a third reviewer was consulted. This third reviewer provided an independent assessment, and the overall mean app quality score was determined by averaging the 2 closest scores among the 3 reviewers.

The MARS was utilized to assess the quality of the apps identified in searches conducted in October 2022 and 2023. For apps identified in both searches, a separate evaluation was conducted after each search if there had been an update to the app. This approach was adopted to examine the changes implemented and to determine whether there was a variation in the app’s quality.

### Ethical Considerations

This study did not involve human participants or personal data collection. According to local policies, ethics approval was not required for this research.

## Results

### Description

**Figure 1.**
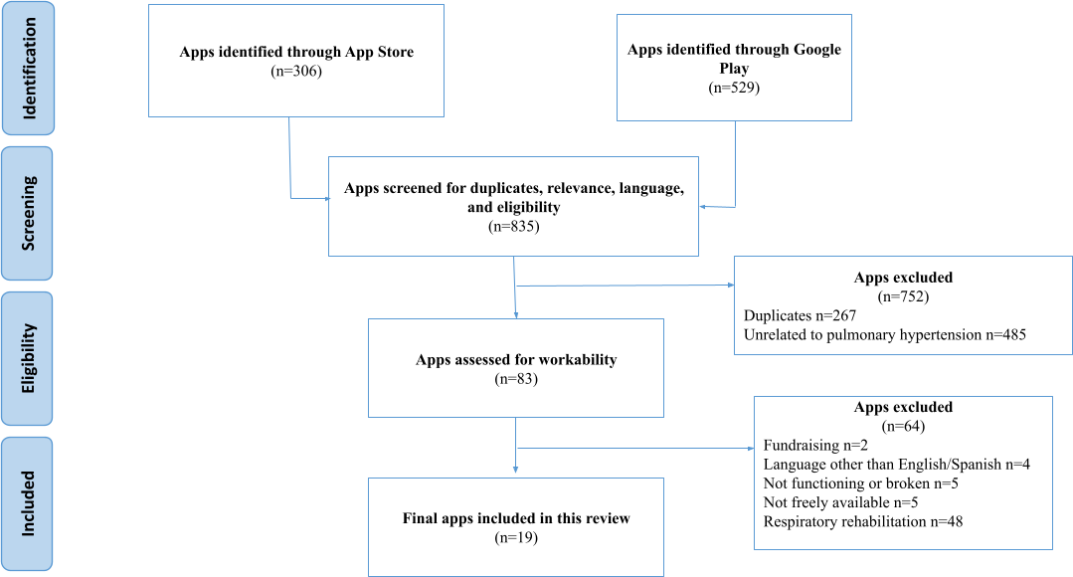
PRISMA flow diagram illustrating the initial search conducted in 2022. PRISMA: Preferred Reporting Items for Systematic Reviews and Meta-Analyses.

In the scope of this study, searches conducted in October 2022 and October 2023 resulted in the cumulative identification of 21 apps. The initial search in 2022 identified 19 apps, from a total of 835 apps screened (Figure 1). Of these 19 apps, only 7 received version updates during 2022.

The subsequent search in 2023 identified 16 apps, of which 14 had been initially identified in the 2022 search and remained available. Only 7 of these apps had received version updates in 2023, as compared to their status after the first search in 2022. Moreover, the 2023 search revealed the introduction of 2 new apps unavailable in the preceding year. Table 1 provides detailed general characteristics of the apps included in this analysis.

Almost half of the assessed apps received funding from the pharmaceutical industry (10/21, 48%), and 4 (19%) were developed by the pharmaceutical industry. This influence was observed mostly in the iOS platform apps.

The primary objective of most of the evaluated apps (17/21; 81%) was to provide general information about PH, as well as other pulmonary pathologies in some instances (n=8; 38%). Of the apps targeted at health care professionals, 10 allowed patient assessments through the use of calculators, facilitating the evaluation/classification of patients. There were 2 apps (6 min. test and Pulmonary hypertension) that provided information and tools to assess pediatric patients with PH. None of the apps facilitated the recording of clinical parameters for patient monitoring. Regarding the apps intended for patient use, none permitted users to register activities or contact health care professionals.

For the apps found in the 2022 search, health care professionals contributed to the development or design of 13 of 19 apps (68%). By the 2023 search, health care professionals were involved in 11 of 16 apps (69%). Regarding patient engagement in the development or design process of these apps, such details were not explicitly stated in the descriptions of any evaluated apps. However, the apps “phaware” and “phaware: Aware That I’m Rare,” developed by Phaware Global Association (a collective of patients, caregivers, and medical professionals), suggest a potential for patient involvement in designing these apps. Table 2 describes the main characteristics of the assessed apps.

**Table 1.** General characteristics of apps.

App		Year			Platform		Language	
Name	Developer	Search	Publication	Last update	iOS	Android	English	Spanish
6 min.test	PH Austria - Lunghochdruck	2022/23	2020	2020	X		X	X
6 Minute Walker	Appricode	2022/23	2021	2021		X	X	X
ATS journals App	American Thoracic Society Inc	2022/23	2013	2021	X		X	
Detect PAH	Janssen EMEA	2022	2022	2022	X		X	
Dx3D	Merck Sharp & Dohme Corp.	2022/23	2021	2023	X		X	
Echoright/Echoright Pro^a^	Janssen EMEA	2022/23	2019	2023	X	X	X	
ePulmonology Review	DKBmed LLC	2022/23	2018	2020	X	X	X	
ESC Pocket Guidelines	ESC European	2022/23	2016	2023	X	X	X	
Hipertensión pulmonar	Goodstore	2022	2021	2021		X		X
Las enfermedades respiratorias	DevoDreamTeam	2022/23	2020	2023		X	X	X
Lung Diseases and Treatment	StudySpring	2023	2023	2023		X	X	
MSD PH art	Merck Sharp & Dohme LLC	2022/23	2022	2023	X		X	
Phaware: Aware That I’m Rare	Phaware global association	2022/23	2016	2023	X		X	
Phaware	Phaware global association	2022/23	2014	2023	X		X	
Pulmonary & Diseases	Medico_guide	2022	2018	2021		X	X	
Pulmonary hypertension	Corey Chartan	2022/23	2018	2018	X		X	
Pulmonary hypertension support	MyHealthTeams	2022	2018	2019	X		X	
Respiratory Diseases and Treatments	Xstream Apps	2022	2020	2020		X	X	
Respiratory Diseases Treatment	Medi Science	2023	2023	2023		X	X	
Respiratory Disease & Treatment	Patrikat Softech	2022/23	2018	2022		X	X	
Respiratory diseases & Treatment	KIFEDHA APP	2022/23	2020	2021		X	X	

a Echoright Pro is the new version (2023) of the Echoright app (2022).

**Table 2.** Characteristics of the assessed apps.

Characteristics	2022 search (n=19), n (%)	2023 search (n=16), n (%)	Total (n=21), n (%)
**Target audience**			
Health professional	9 (47)	8 (50)	9 (43)
Patients/general population	10 (53)	8 (50)	12 (57)
**Contain advertisements**	7 (37)	6 (38)	9 (43)
**Industry-funded** ^a^	10 (53)	8 (50)	10 (48)
**Type of developer**			
Commercial/private companies	9 (47)	7 (44)	11 (52)
Scientific/patient society/nongovernmental organization	6 (32)	6 (38)	6 (29)
Pharmaceutical industry	4 (21)	3 (19)	4 (19)
**Age group**			
All audiences	5 (26)	5 (31)	7 (44)
>4 years	4 (21)	4 (25)	4 (19)
>12 years	5 (26)	4 (25)	5 (24)
>17 years	5 (26)	3 (19)	5 (24)
**Technical aspects of apps**			
Allows sharing (Facebook, Twitter, etc)	13 (68)	10 (62)	15 (71)
Requires login	8 (42)	6 (38)	8 (38)
Needs web access to function	9 (48)	7 (44)	9 (43)

a Apps that have received funding or sponsorship from the pharmaceutical industry, irrespective of whether the developer is the pharmaceutical industry or not.

### Assessment of App Quality

The overall MARS app quality was considered acceptable in both 2022 and 2023, with mean scores of 3.1 (SD 0.6) and 3.3 (SD 0.5), respectively. Table 3 presents the collected MARS overall quality scores for reference.

The quality assessment of the apps, as detailed in Multimedia Appendices 1 and 2, presents the mean and standard deviations for each MARS item score for the 2022 and 2023 searches, respectively. The engagement scores exhibited significant variation across the apps. Notably, 58% (11/19) of the apps in 2022 and 56% (9/16) in 2023 scored below 3, with the lowest ratings observed in customization and interactivity for both years. In contrast, the functionality category received the highest ratings, achieving a consistent score of 3.8 in both 2022 and 2023. A notable 53% (n=10) of apps in 2022 and 69% (n=11) in 2023 were evaluated with scores of 4 or higher, reflecting commendable performance characterized by ease of use and intuitive navigation. Aesthetically, all evaluated apps received favorable ratings, attributed to their well-designed layouts, graphical design, and visual appeal.

In terms of informational content, most of the apps offered clear descriptions of their features in the app store. However, a significant portion lacked specific objectives, with 47% (n=9) in 2022 and 75% (n=12) in 2023 falling into this category. Evaluations generally favored the quality of the information provided over the quantity, indicating a preference for well-articulated and purposeful content rather than a larger volume of less specific information.

In terms of subjective quality, this domain was the lowest rated in the MARS assessment for both years, with a score of 1.9. Approximately 63% (n=12) of the apps in 2022 and 50% (n=8) in 2023 received a score of 2 or lower, indicating that they were not highly recommendable. Furthermore, 42% (n=8) of the apps in 2022 and 44% (n=7) in 2023 achieved a 3-point score for frequency of use. Notably, when responding to the MARS subjective quality item “Would you pay for this app?” none of the evaluators found the apps worthy of purchase.

**Table 3.** The Mobile Application Rating Scale overall quality scores.

App name and year		Engagement, mean (SD)	Functionality, mean (SD)	Aesthetics, mean (SD)	Information, mean (SD)	Subjective quality, mean (SD)	Overall score, mean (SD)
**6 min.test**							
2022/23^a^		2.4 (0.5)	4.0 (0)	3.3 (0.6)	3.2 (0.8)	1.8 (0.5)	3.2 (0.7)
**6 Minute Walker**							
2022/23^a^		2.6 (1.1)	4.0 (0)	3.0 (0)	2.4 (0.9)	2.0 (0.8)	3.0 (0.7)
**ATS journals APP**							
2022/23^a^		3.2 (1.1)	4.0 (0)	3.3 (0.6)	4.2 (0.4)	2.5 (1.0)	3.7 (0.5)
**Detect PAH**							
2022		2.4 (1.1)	3.8 (0.5)	3.3 (0.6)	2.8 (0.8)	1.5 (0.6)	3.1 (0.6)
**Dx3D**							
2022		3 (1.4)	3.8 (0.5)	3.7 (0.6)	3.7 (0.5)	2.3 (1)	3.52 (0.3)
2023		3.2 (1.1)	4.0 (0)	4.0 (0)	3.7 (0.5)	2.3 (1)	3.7 (0.4)
**Echoright/Echoright Pro** ^**b**^							
2022		3.2 (1.3)	4.0 (0)	4.0 (0)	3.7 (0.5)	2.3 (1)	3.72 (0.4)
2023		3.2 (1.3)	4.0 (0)	4.0 (0)	3.8 (1)	2.3 (1)	3.8 (0.4)
**ePulmonology Review**							
2022/23^a^		2.8 (1.3)	4.0 (0)	4.0 (0)	3.4 (0.5)	2.3 (1)	3.55 (0.6)
**ESC Pocket Guidelines**							
2022		3.2 (1.3)	4.3 (0.5)	3.0 (0)	3.0 (0)	2 (0.8)	3.4 (0.6)
2023		3.4 (1.1)	4.3 (0.5)	4.0 (0)	3.6 (0.9)	2.5 (1)	3.8 (0.4)
**Hipertensión pulmonar**							
2022		1.8 (0.8)	3.5 (0.6)	2.0 (0)	2.2 (0.8)	1.3 (0.5)	2.4 (0.8)
**Las enfermedades respiratorias**							
2022		2.6 (0.5)	3.8 (0.5)	2.7 (0.6)	3.2 (1.3)	1.5 (0.6)	3.1 (0.5)
2023		2.8 (0.4)	3.8 (0.5)	3.0 (0)	2.8 (1.1)	1.5 (0.6)	3.1 (0.5)
**Lung Diseases and Treatment**							
2023		2.2 (1.1)	4.0 (0)	3.7 (0.6)	3.0 (1.2)	1.5 (0.6)	3.2 (0.8)
**MSD PH art**							
2022		3.2 (1.3)	4.0 (0)	4.0 (0)	3.6 (0.5)	3 (1.4)	3.7 (0.4)
2023		3.2 (0.8)	4.3 (0.5)	4.0 (0)	3.5 (1.3)	2.5 (1)	3.7 (0.5)
**phaware**							
2022/23^a^		2.8 (0.8)	3.3 (0.5)	3.0 (0)	4.0 (1.4)	2.0 (0.8)	3.3 (0.5)
**phaware: Aware That I´m Rare**							
2022/23^a^		3.2 (0.4)	4.0 (0)	3.7 (0.6)	4.0 (0)	2.3 (1)	3.7 (0.4)
**Pulmonary hypertension**							
2022/23^a^		3 (1.4)	4.8 (0.5)	3.7 (0.6)	3.2 (0.8)	2.3 (1)	3.7 (0.8)
**Pulmonary hypertension support**							
2022		3.4 (0.9)	3.8 (0.5)	3.7 (0.6)	3.6 (0.5)	2.5 (1)	3.6 (0.1)
**Pulmonary & Diseases**							
2022		1.6 (0.5)	3.5 (1)	1.7 (0.6)	1.7 (0.8)	1.0 (0)	2.1 (0.9)
**Respiratory Diseases and Treatments**							
2022		2.2 (1.1)	4.0 (0)	2.7 (0.6)	2.7 (1)	1.5 (0.6)	2.9 (0.8)
**Respiratory Diseases Treatment**							
2023		1.6 (0.5)	3.5 (0.6)	3.3 (0.6)	2.4 (0.9)	1.0 (0)	2.7 (0.9)
**Respiratory Disease & Treatment**							
2022		1.2 (0.4)	2.5 (0.6)	1.7 (0.6)	1.8 (0.8)	1 (0)	1.8 (0.5)
2023		1.8 (0.8)	2.8 (0.5)	2.7 (0.6)	2.6 (0.4)	1.3 (0.5)	2.5 (0.4)
**Respiratory diseases & Treatment**							
2022/23^a^		1.2 (0.4)	3.0 (0)	2.0 (0)	1.8 (0.8)	1.0 (0)	2.0 (0.7)
**Total, mean (SD)**							
2022		2.58 (0.7)	3.8 (0.5)	3.1 (0.8)	3.1 (0.8)	1.9 (0.6)	3.1 (0.6)
2023		2.7 (0.7)	3.8 (0.5)	3.4 (0.6)	3.2 (0.7)	1.9 (0.5)	3.3 (0.5)

a The app remained available in both years; however, a reevaluation was not conducted due to the absence of any updates since the initial assessment.

b Echoright Pro is the new version (2023) of the Echoright app (2022).

### Evolution of Apps Across Two Years

The mean overall MARS score exhibited a slight improvement from 2022 to 2023 (+0.2). However, this variation could be considered not statistically significant, suggesting overall quality remained stable across the evaluated years.

**Figure 2.**
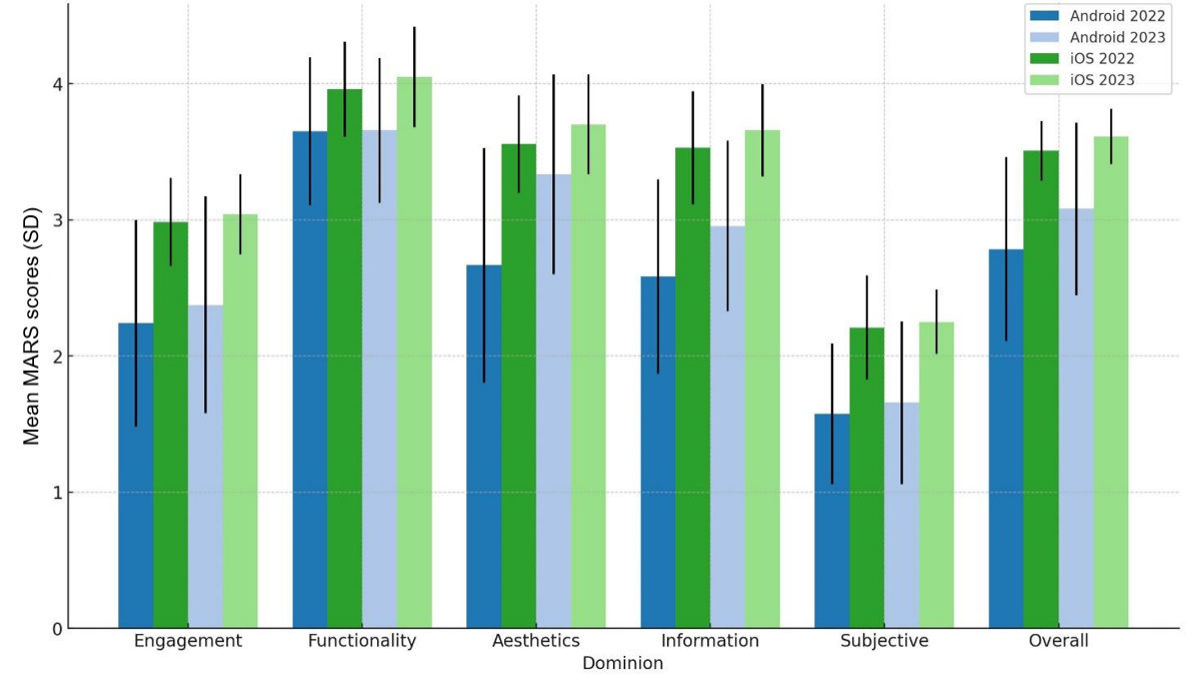
Quality differences between platforms across the 2-year evaluations.

Multimedia Appendix 2 details the differences in scores based on the evaluated platform. Specifically, iOS apps consistently received higher ratings compared to Android apps in terms of the overall mean MARS score for both years, with a difference of +0.7 (*P*=.008) in 2022 and +0.5 (*P*=.03) in 2023. The 2022 search revealed statistically significant differences in the domains of engagement (+0.8, *P*=.02), aesthetics (+0.9, *P*=.01), information (+0.9, *P*=.005), and subjective quality (+0.6, *P*=.008). In contrast, the 2023 search found significant differences only in the information (+0.8, *P*=.01) and subjective quality (+0.6, *P*=.03) domains. Figure 2 illustrates these variations in each MARS domain across the 2 years (2022‐2023).

Regarding the involvement of health care professionals, apps that incorporated their input in design or development showed higher overall MARS ratings in both the 2022 (+1.1, *P*<.001) and 2023 (+0.9, *P*=.002) searches. Enhanced ratings were observed across all 5 domains in both searches, as detailed in Multimedia Appendix 3.

## Discussion

### Main Results

Health apps have the potential to become a standard component of care for individuals with chronic conditions. These apps have a demonstrated beneficial impact on patients’ health, including enhanced adherence to prescribed medications, improved quality of life, and reduced reliance on health care services [16,30-32]. To our knowledge, this is the first study that systematically evaluated and compared the quality of free mHealth apps aimed at patients with PH and health care professionals over 2 years using a cross-sectional approach, with the aim of examining the progression in the quality and features of PH apps.

In this systematic evaluation, 21 unique PH apps were identified, including 19 in 2022 and 16 in 2023. The overall mean app quality score improved from 3.1 (SD 0.6) in 2022 to 3.3 (SD 0.5) in 2023. According to the MARS 5-point scale, a score above 3, which denotes “acceptable” quality, generally indicates that an app is functional and offers a satisfactory user experience but may still lack certain advanced features or innovative design elements that could elevate its rating to “excellent.” However, it is important to note that the MARS framework does not provide explicit guidelines on interpreting the overall mean app quality total score, leaving some ambiguity in assessing app quality [24,25]. Therefore, we acknowledge that for health care apps, especially for complex conditions like PH, more stringent quality standards for “acceptable” quality could be necessary, such as a cutoff score of 3.5 (indicating above-average quality), as defined by Terhorst et al, which may better reflect the standards needed for effective health care management apps [33].

Throughout the 2-year study, from the 19 apps initially identified in 2022, only 14 remained available in 2023. Notably, these persisting apps scored above the median on the MARS, suggesting that higher quality may have contributed to their continued availability in the market. The decrease in availability of the remaining apps calls for further investigation. Potential reasons for this decline could include low overall quality, poor user engagement, lack of economic viability, regulatory challenges, obsolescence in content or technology, organizational changes, or negative performance and reviews [34-36]. In contrast, most apps that remained available over the 2-year period showed durability, although with a relatively low frequency of updates. The minimal changes observed, even after updates, indicate a lack of significant advancement over the year. This might indicate a continuing interest in the digital management of PH but also points to a potential lack of advancement, a significant issue in the fast-evolving mHealth sector [37]. The limited updates could result from funding constraints or inadequate user feedback, both critical for driving innovation in this area.

Simultaneously, the involvement of health care professionals in creating mHealth apps emerged as a crucial factor influencing both the quality and the accuracy of the information provided [17,28,38]. A systematic analysis focusing on the contribution of experts and their compliance with medical evidence in mHealth apps revealed a notable trend: many health apps lack health care professional input and are not consistently grounded in evidence-based information [38]. This is noteworthy considering that the involvement of health care experts in the app development process does not automatically guarantee the inclusion of evidence-based content [38,39]. However, in the context of PH, our review found that more than two-thirds of PH-related apps were developed with the active involvement of health care professionals, correlating with higher app quality scores (2022: 13/19, 68%; 2023: 11/16, 69%). These findings suggest that health care professionals are a key part in developing high-quality PH-related apps.

Moreover, specific app functionalities exhibit significant potential for health care professionals managing PH. Key features such as pulmonary arterial pressure monitoring for patients with implanted devices and easy access to up-to-date PH-specific treatment guidelines are distinctly advantageous [40,41]. Within the scope of this review, 2 apps provided access to PH-specific treatment guidelines (“ESC pocket guidelines” and “ATS journals APP”), and 10 apps included risk assessment tools. However, a critical issue is the infrequent updates; only half of the apps from the year 2022 were updated that year, and just 43% of those available in 2023 had received recent updates, underscoring a significant gap in maintaining current information.

Mobile apps targeted at patients with PH could profoundly impact health outcomes by enhancing self-management, monitoring, and communication [16,30,42]. Such apps could aid patients with PH through various functionalities like symptom tracking, ensuring medication adherence, enabling remote monitoring, offering patient education, creating support networks, facilitating telemedicine, and providing mental health support [43-45]. However, our findings reveal that the majority of apps lack direct patient engagement in their design, resulting in the omission of key features like direct contact with health care professionals and patient activity registration. Notably, only 2 apps, “Pulmonary Hypertension Support” and “phaware,” provided community access, with the latter doing so through external links. This gap highlights the need for greater patient involvement in app development to ensure the tools meet the actual needs of end users, potentially through user-centered design approaches [46,47]. The apparent mismatch between app functionalities and patient needs suggests an area ripe for research, aimed at aligning app development more closely with patient priorities [48-50].

A significant finding from our systematic evaluation is the absence of proven clinical efficacy among PH apps, highlighted by the fact that none underwent formal testing in clinical trials for their efficacy in PH management—a stark contrast to chronic conditions like heart failure or chronic obstructive pulmonary disease, where mHealth interventions have demonstrated clinical benefits [51-54]. Notably, apps like “Detect PAH” by Janssen EMEA, which includes a clinically validated diagnostic algorithm [55-57], and the “6 min.test” app by PH Austria, featuring a pediatric-adapted 6-minute walking distance test [58-60], showed promise but lacked validation in broader clinical settings. This lack of broader clinical validation is consistent with findings from other studies assessing health apps [17,28,61]. Further research is crucial to comprehensively evaluate these apps prior to their integration into standard PH management, considering the potential for eHealth to add to the treatment burden, especially for vulnerable patients [62,63].

### Additional Considerations

Beyond the limitations identified in this systematic evaluation, free applications for patients with PH and caregivers should also focus on enhancing interoperability and ensuring robust data privacy.

Enhancing interoperability with existing health care systems is essential to maximize the utility of free health apps. Interoperability ensures seamless communication with other health information systems, which is crucial for real-time updates and integration of the latest treatment guidelines and diagnostic tools. This connectivity keeps apps relevant and effective for both patients and health care providers [64]. By adhering to established health data standards such as Health Level 7 [65] and Fast Healthcare Interoperability Resources [66], these free apps can improve their adaptability and usefulness, facilitating better clinical decision-making and improving patient outcomes [51-54].

However, free apps often pose significant privacy and security risks. According to a report by Timeero [67], free apps tend to share more user data compared to paid apps, increasing the risk of data breaches and unauthorized access. The report indicates that free apps share, on average, 7 times more data points than their paid counterparts, often monetizing user data by selling it to third parties. This practice raises substantial concerns, especially given the sensitive nature of health data. App developers need to implement robust data protection measures to safeguard patient information. This involves encryption of data both at rest and in transit, secure user authentication mechanisms, and regular security audits to prevent unauthorized access and data breaches [68]. Additionally, compliance with regulatory requirements such as the General Data Protection Regulation [69] in Europe or the Health Insurance Portability and Accountability Act [70] in the United States is critical. For free apps, which may lack the financial backing of paid apps, ensuring these privacy and security measures can be challenging but is necessary to gain user trust and ensure safe use in a health care setting.

### Limitations

This systematic evaluation is subject to limitations. The evaluation process utilizes the MARS framework [24,26], which introduces a degree of subjectivity in app categorization. However, the dual-reviewer methodology applied in this study likely reduces the impact of this subjectivity. In addition, the MARS framework, while being a widely recognized tool for app quality assessment [17,27,28,71], does not account for crucial factors such as privacy, security, and update frequency, which are critical for comprehensive health software quality assessment [72,73].

In addition, our perspective as health care professionals may influence our interpretation of patient-oriented apps’ relevance and utility for patients, potentially overlooking user-specific needs and preferences [49,50]. Furthermore, our analysis was primarily based on app store descriptions and publicly available information, which limits our ability to verify the extent or nature of health care professional or patient involvement in the development of these apps. Additionally, the methodology involved systematic searches conducted via mobile devices in order to simulate the experience of app users. This approach potentially introduces bias based on app store search algorithms and the variable availability of apps. Finally, the review was confined to apps available on Google Play and the App Store. Although these are the major platforms covering most market-available apps [74], other platforms such as Windows Phone and Blackberry Market were not included due to the unavailability of devices for the reviewers, which may have led to the omission of certain PH-related apps.

### Conclusions

This systematic evaluation of PH-related mHealth apps targeted at patients and health care professionals has provided critical insights into the current state and potential of these tools. Our analysis identified a relatively small cohort of apps, with an average quality score deemed acceptable, yet revealing a considerable gap in the development and refinement of these digital resources. Furthermore, the involvement of health care professionals in the design and development of mHealth apps was found to be a significant factor in enhancing the quality and reliability of the information these tools provide. Despite this positive impact, our study observed a stagnation in the evolution of app quality and features over 2 years, highlighting a critical need for continuous innovation and improvement. Additionally, our findings emphasize the lack of clinical validation for most of these apps, which is a pivotal step that should not be overlooked if these tools are to be integrated into standard patient care.

In conclusion, while the landscape of mHealth apps for PH shows promise, it remains underdeveloped. Future efforts should focus on leveraging the expertise of health care professionals, integrating patient perspectives, and ensuring that apps undergo rigorous clinical validation. Such measures would significantly enhance the utility, relevance, and effectiveness of mHealth tools, potentially transforming them into indispensable components of PH management. This evolution requires a concerted effort from stakeholders to realize the full potential of mHealth in supporting the complex needs of patients with PH.

## Supplementary material

10.2196/57289 Multimedia Appendix 1 The Mobile Application Rating Scale—engagement, functionality, aesthetics, and information domains (2022 search).

10.2196/57289 Multimedia Appendix 2 Differences between platform scores.

10.2196/57289 Multimedia Appendix 3 Differences between health care professionals’ participation in the apps’ development.

10.2196/57289 Multimedia Appendix 4 The Mobile Application Rating Scale—engagement, functionality, aesthetics, and information domains (2023 search).

## Abbreviations

MARS: Mobile Application Rating Scale
mHealth: mobile health
PH: pulmonary hypertension
