# Supplementary material for: Smartphone Apps for Pulmonary Hypertension: Systematic Search and Content Evaluation
Source: JMIR Mhealth Uhealth. 2024 Oct 30;12:e57289. doi: 10.2196/57289 (PMC11540248; doi:10.2196/57289)
Supplement: Multimedia Appendix 1 [file mhealth-v12-e57289-s001.docx]

**Multimedia appendix 1**. The Mobile Application Rating Scale – engagement, functionality, aesthetics and information domains- 2022 search.

| Name | **Engagement score** | | | | | **Functionality score** | | | | **Aesthetics score** | | | **Information score** | | | | | |
| --- | --- | --- | --- | --- | --- | --- | --- | --- | --- | --- | --- | --- | --- | --- | --- | --- | --- | --- |
|  | Ent | Int | Cus | Ly | Tg | Per | EU | Nav | GD | Lay | Gra | VA | AAD | Goa | QI | QyI | VI | Cre |
|  |  |  |  |  |  |  |  |  |  |  |  |  |  |  |  |  |  |  |
| 6 min.test | 2 | 3 | 2 | 2 | 3 | 4 | 4 | 4 | 4 | 4 | 3 | 3 | 4 | ND | 4 | 3 | 2 | 3 |
| 6 Minute Walker | 4 | 3 | 1 | 2 | 3 | 4 | 4 | 4 | 4 | 3 | 3 | 3 | 4 | 2 | 2 | 2 | ND | 2 |
| ATS journals APP | 4 | 4 | 2 | 2 | 4 | 4 | 4 | 4 | 4 | 4 | 3 | 3 | 5 | 4 | 4 | 4 | 4 | 4 |
| Detect PAH | 2 | 3 | 1 | 2 | 4 | 4 | 4 | 4 | 3 | 4 | 3 | 3 | 3 | 4 | 3 | 2 | 3 | 2 |
| Dx3D | 4 | 4 | 1 | 2 | 4 | 4 | 4 | 4 | 3 | 4 | 4 | 3 | 4 | 3 | 4 | 3 | 4 | 4 |
| Echoright | 4 | 4 | 1 | 3 | 4 | 4 | 4 | 4 | 4 | 4 | 4 | 4 | 4 | 4 | 3 | 4 | 4 | 3 |
| ePulmonology Review | 3 | 4 | 1 | 2 | 4 | 4 | 4 | 4 | 4 | 4 | 4 | 4 | 4 | ND | 4 | 3 | 3 | 3 |
| Esc Pocket Guidelines | 3 | 4 | 2 | 2 | 5 | 4 | 5 | 4 | 4 | 3 | 3 | 3 | 3 | ND | 3 | 3 | 3 | 3 |
| Hipertensión pulmonar | 3 | 2 | 1 | 1 | 2 | 3 | 4 | 4 | 3 | 2 | 2 | 2 | 3 | 1 | 2 | 2 | 3 | 2 |
| Las enfermedades respiratorias | 3 | 3 | 2 | 2 | 3 | 3 | 4 | 4 | 4 | 3 | 2 | 3 | 4 | ND | 4 | 3 | 4 | 1 |
| MSD PH art | 4 | 4 | 1 | 3 | 4 | 4 | 4 | 4 | 4 | 4 | 4 | 4 | 4 | ND | 4 | 3 | 4 | 3 |
| phaware | 3 | 3 | 2 | 2 | 4 | 3 | 4 | 3 | 3 | 3 | 3 | 3 | 4 | ND | 4 | ND | ND | ND |
| phaware: Aware That I´m Rare | 4 | 3 | 3 | 3 | 4 | 4 | 4 | 4 | 4 | 4 | 4 | 3 | 4 | ND | 4 | ND | ND | ND |
| Pulmonary & Diseases | 2 | 2 | 1 | 1 | 2 | 4 | 4 | 4 | 2 | 2 | 2 | 1 | 3 | 1 | 2 | 1 | 1 | 2 |
| Pulmonary hypertension | 4 | 4 | 1 | 2 | 4 | 4 | 5 | 5 | 5 | 4 | 4 | 3 | 4 | ND | 4 | 3 | 3 | 2 |
| Pulmonary hypertension support | 4 | 4 | 2 | 3 | 4 | 4 | 4 | 4 | 3 | 4 | 4 | 3 | 4 | ND | 3 | 4 | 4 | 3 |
| Respiratory Diseases and Treatments | 3 | 3 | 1 | 1 | 3 | 4 | 4 | 4 | 4 | 3 | 2 | 3 | 4 | 1 | 3 | 3 | 3 | 2 |
| Respiratory Disease &Treatment | 1 | 1 | 1 | 1 | 2 | 2 | 3 | 3 | 2 | 2 | 2 | 1 | 3 | 1 | 2 | 2 | 2 | 1 |
| Respiratory diseases&Treatment | 1 | 1 | 1 | 1 | 2 | 3 | 3 | 3 | 3 | 2 | 2 | 2 | 3 | 1 | 2 | 2 | 2 | 1 |
| **Mean, SD** | 3.11 (1) | 3.1 (1.1) | 1.4 (0.6) | 2.0 (0.7) | 3.4 (0.9) | 3.7 (0.6) | 4.0 (0.5) | 3.9 (0.5) | 3.5 (0.8) | 3.3 (0.8) | 3.1 (0.8) | 2.8 (0.8) | 3.7 (0.6) | 2.2 (1.5) | 3.2 (0.9) | 2.8 (1.2) | 3.1 (1.4) | 2.4 (1.2) |

AAD: accuracy of app description, Cre: credibility, Cus: customization, Ent: entertainment, EU: ease of use, GD: gestural design, Goa: goals, Gra: graphics, Int: interest, Iy: interactivity, Lay: layouts, N/A: not applicable, Nav: navigation, Per: performance, QI: quality of information, QyI: quantity of information, Tg: target group, VA: visual appeal, VI: visual information.
