# Supplementary material for: Smartphone Apps for Pulmonary Hypertension: Systematic Search and Content Evaluation
Source: JMIR Mhealth Uhealth. 2024 Oct 30;12:e57289. doi: 10.2196/57289 (PMC11540248; doi:10.2196/57289)
Supplement: Multimedia Appendix 2 [file mhealth-v12-e57289-s002.docx]

**Multimedia appendix 3.** Differences between platform scores

| Year | 2022 | | | 2023 | | |
| --- | --- | --- | --- | --- | --- | --- |
|  | Android (SD) | IOS  (SD) | p | Android (SD) | IOS  (SD) | p |
|  |  |  |  |  |  |  |
| Engagement | 2.2 (0.8) | 3.0 (0.3) | 0.0171* | 2.40 (0.8) | 3.0 (0.3) | 0.0501 |
| Functionality | 3.7 (0.5) | 4.0 (0.4) | 0.2515 | 3.7 (0.5) | 4.1 (0.4) | 0.1017 |
| Aesthetic | 2.7 (0.9) | 3.6 (0.4) | 0.0147* | 3.3 (0.7) | 3.7 (0.4) | 0.2006 |
| Information | 2.6 (0.7) | 3.5 (0.4) | 0.0049* | 2.9 (0.6) | 3.7 (0.3) | 0.0112* |
| Subjective quality | 1.6 (0.5) | 2.2 (0.4) | 0.0078* | 1.7 (0.6) | 2.3 (0.3) | 0.0317* |
| Overall APP | 2.5 (0.6) | 3.3 (0.3) | 0.0075* | 3.1 (0.6) | 3.6 (0.2) | 0.0497* |

SD: Standard deviation; P: Mann-Whitney U test.
