# Supplementary material for: Smartphone Apps for Pulmonary Hypertension: Systematic Search and Content Evaluation
Source: JMIR Mhealth Uhealth. 2024 Oct 30;12:e57289. doi: 10.2196/57289 (PMC11540248; doi:10.2196/57289)
Supplement: Multimedia Appendix 3 [file mhealth-v12-e57289-s003.docx]

**Multimedia appendix 4**. Differences between healthcare professionals' participation in app’s development

| Year | 2022 | | | 2023 | | |
| --- | --- | --- | --- | --- | --- | --- |
|  | HCP-P | HCP-NP | p | HCP-P | HCP-NP | p |
|  |  |  |  |  |  |  |
| Engagement | 3.0 (0.3) | 1.8 (0.5) | 0.0014* | 3.00 (0.3) | 1.9 (0.6) | 0.0056* |
| Functionality | 4.0 (0.3) | 3.4 (0.5) | 0.0152* | 4.1 (0.4) | 3.4 (0.5) | 0.0130* |
| Aesthetic | 3.5 (0.4) | 2.1 (0.4) | 0.0006* | 3.6 (0.4) | 2.9 (0.6) | 0.0367* |
| Information | 3.4 (0.5) | 2.2 (0.5) | 0.0031* | 3.5 (0.5) | 2.6 (0.4) | 0.0076* |
| Subjective quality | 2.2 (0.4) | 1.2 (0.2) | 0.0008* | 2.3 (0.3) | 1.3 (0.3) | 0.0018* |
| Overall APP | 3.5 (0.3) | 2.4 (0.5) | 0.0001* | 3.6 (0.3) | 2.7 (0.5) | 0.0018* |

HCP-P: Healthcare professionals’ participation; HCP-NP: Healthcare professional's no participation; p: Mann-Whitney U test; SD: Standard deviation.
